# Critical care utilisation, and outcomes of critically ill obstetric patients in India: a multicentre ICU registry study (2019–2024)

**DOI:** 10.1016/j.lansea.2026.100845

**Published:** 2026-08-26

**Authors:** Swagata Tripathy, Dilanthi Gamage Dona, Bharath Kumar Tirupakuzhi Vijayaraghavan, Neha Singh, Sweta Singh, Rashan Haniffa, Abi Beane, Luigi Pisani

**Affiliations:** a AIIMS Bhubaneswar, India; b Nat Intensive Care Surveillance- MORU, Colombo, Sri Lanka; c Department of Critical Care Medicine, Apollo Hospitals, Chennai, India; d Centre for Inflammation Research, University of Edinburgh, United Kingdom; e Department of Precision-Regenerative Medicine and Jonic Area (DiMePRe-J), Section of Anesthesiology and Intensive Care Medicine, University of Bari “Aldo Moro”, Bari, Italy; f Mahidol Oxford Tropical Research Unit (MORU), Bangkok, Thailand

**Keywords:** Obstetric critical care, Outcomes, Clinical quality databases, ICU registry, Severity of illness, LMICs

## Abstract

**Background:**

We aimed to characterise the case mix, severity scores, organ support interventions and outcomes of obstetric patients admitted to 40 ICUs in India over a 5 year period.

**Methods:**

We analysed prospectively collected data from the Indian Registry of IntenSive care (IRIS) including female patients aged 15–49 years admitted between January 2019 and November 2024. Obstetric patients were compared with non-obstetric female ICU patients. The obstetric cohort was further stratified by surgical and hospital status.

**Findings:**

Obstetric patients constituted 5.8% (577/10006) of women of reproductive age admitted to 40 ICUs, of which 9 were public. These women were younger (median 29 versus 37 years), more often admitted after surgery, with similar acuity of illness, organ support use and ICU length of stay–but lower ICU mortality (3.3% versus 7.9%, Absolute standardised Difference ASD = 0.2) than the non-obstetric cohort. Within the obstetric patients, non-operative patients required less organ support but longer ICU stays. Patients admitted to public ICUs had higher illness severity at admission (median APACHE II score 12 versus 8, p = 0.001), higher crude ICU mortality (6.4% versus 2.1%, p = 0.02), albeit with comparable standardised mortality rates.

**Interpretation:**

Critically ill obstetric patients present with distinct clinical trajectories and outcomes. APACHE II score overestimated mortality in obstetric patients. Public ICUs face higher illness severity and worse crude mortality, but comparable risk-adjusted outcomes to private ICUs. Registries provide a scalable platform to support evidence-based maternal critical care planning in India.

**Funding:**

The IRIS registry is part-funded by the Wellcome Trust.

**Research in context**

**Evidence before this study**

A meta-analysis of critical care admissions and outcomes of peripartum patients, we performed in 2024 reported data from 71 studies and over 111,000 ICU admissions found substantial global heterogeneity in case definitions, ICU admission criteria, and reported outcomes. Overall obstetrics ICU admissions were low (1.6%) but significantly higher in LMICs (low and middle income countries) than HICs (high income countries) (2.8% versus 0.4%, p < 0.0001). ICU mortality showed an even larger disparity, with LMICs reporting nearly nine-fold higher mortality than HICs (12.4% versus 1.4%, p < 0.0001). Meta-regression confirmed geographic income status as a strong predictor of both ICU admission and mortality. Indian ICU obstetric data was derived from 15 small-to-moderate single-centre studies and demonstrated a wide variability in sample size and heterogeneous reporting formats—justifying a contemporary, larger analysis of more uniform datasets from India.

**Added value of this study**

This study presents prospectively collected data over 5 years from 40 public and private ICUs across India. It presents a direct comparison between obstetric and non-obstetric critically ill women of the reproductive group, within the obstetric cohort and different healthcare systems, using standardised nomenclature, illness severity and outcome metrics. While three out of 100 patients still do not survive ICU discharge, we demonstrate the persistent divergence between APACHE 2-predicted versus observed mortality in obstetric patients. We also document distinct surgical versus medical trajectories and a significant use of short-stay postoperative ICU utilisation. Of note, we underline the importance of using risk-adjusted outcomes, as public hospitals were burdened with higher severity of illness and crude mortality despite a comparable risk-adjusted performance to private institutions.

**Implications of all the available evidence.**

Obstetric ICU care still represents a core service in public and private ICUs in India and other LMICs, with gradual improvements in outcome. Strengthening system-level ICU capacity and pre-ICU referral pathways while calibrating prognostic tools to better reflect obstetric physiology may improve risk stratification and resource allocation–potentially impacting maternal critical care outcomes. Registry-enabled analyses allow robust ICU epidemiological surveillance and patient profiling, while interventional studies may focus on delineating targets for improving triage, resource allocation and utilisation across patient care pathways.

## Introduction

Improving maternal and child health remains a cornerstone of the global health agenda and is prominently featured in the Sustainable Development Goals (SDGs) proposed by the World Health Organisation.^1^^,^^2^ Although substantial progress has been made in reducing maternal mortality rates over the past two decades, the burden of pregnancy-related acute and critical illness remains disproportionately high in low- and middle-income countries (LMICs) such as India, which continue to account for the vast majority of global maternal deaths.^3^, ^4^, ^5^ The most common underlying causes (hypertensive disorders of pregnancy, obstetric haemorrhage, and sepsis) frequently require critical care monitoring and interventions. The provision of high-quality intensive care for obstetric patients is essential in mitigating maternal morbidity and mortality.

There has been a scarcity of robust, multicentre data detailing the clinical characteristics, interventions, and outcomes of obstetric patients admitted to intensive care units (ICUs), especially in resource-limited settings.^6^^,^^7^ A recent systematic review and meta-analysis included data from 111,601 women admitted to the intensive care units (ICU) over 41.3 million deliveries reported in 65 studies; however, studies from LMICs (15 from Indian ICUs) had small sample sizes, and methodological heterogeneity with data often sourced from obstetric rather than ICU registries providing limited insights into ICU-specific parameters.^4^

It remains unclear whether critically ill obstetric patients represent merely a younger, healthier subset of general ICU admissions, or a distinct critical illness phenotype with different disease trajectories, resource utilisation, and recovery. There are concerns of commonly used ICU severity-of-illness scores, their calibration and interpretability in obstetric critical care. In addition, how health system context—including public versus private sector care—influences illness severity at ICU admission and subsequent outcomes for obstetric patients in LMICs remains poorly described.

Using data from a, multicentre ICU registry in India, we aimed to characterise the case-mix, severity of illness, organ support, and outcomes of critically ill obstetric patients, and to examine differences in clinical trajectories, prognostic performance, and outcomes across patient subgroups and health system settings. We hypothesized that obstetric critically ill patients constitute a distinct group, with specific features characterising surgical versus medical admissions.

## Methods

We performed an analysis of prospectively collected, multicentre data curated by the Indian Registry of IntenSive care (IRIS), a cloud-based, opt-in clinical quality registry focused on evaluating case-mix and outcomes for critically ill patients at participating hospitals. IRIS covers 26 hospitals and 50 ICUs across India.^8^ All major regions in India were represented, with a majority of hospitals located in southern India. Data for the current analysis was derived from 40 ICUs across 29 hospitals. Registry infrastructure, data quality assurance and governance policies were previously described.^9^^,^^10^ This study is being reported as per the STROBE guidance and the RECORD extension.^11^

Women aged 15–49 years, who were admitted to registry ICUs between January 2019 and November 2024, and coded as pregnant, postpartum or with an obstetric-related diagnosis, were included as ‘obstetric’ admissions. Patients were excluded if aged <15 years or >49 years (as by the WHO definition of reproductive age), readmitted to the ICU during the same hospital admission, or had incomplete ICU discharge information in the registry.^8^^,^^9^^,^^12^ The comparator group was female ICU patients aged 15 to 49 admitted to the ICU during the same period for non-obstetric causes (‘non-obstetric group’). Within the obstetric group, further predefined subgroups were ‘operative’ versus ‘non-operative’ and ‘public’ versus ‘private’, based on the admission status and type of hospital respectively. Operative admissions were defined as ICU admissions immediately following obstetric as well as non-obstetric surgeries for pregnant patients. Pregnancy-related cases were identified using SNOMED/APACHE IV diagnostic terminology and keyword-based extraction of obstetric-related conditions from the registry, followed by clinician validation. Patients documented as pregnant, peripartum, or postpartum were included, along with pregnancy-related complications including miscarriage, ectopic pregnancy, puerperal sepsis, eclampsia and antepartum haemorrhage.

De-identified patient records provided data on demographics, admission diagnostic categories, comorbidities, organ support (mechanical ventilation, vasopressors and renal replacement therapy within the first 24 h of admission), severity of illness scores (APACHE-II), year of ICU admission, source of ICU admission and type of admission (operative versus non-operative) and surgical status (elective or emergency surgery). Information on the healthcare setting (hospital/ICU classification-public or private) was also included. Obstetric-related ICU admissions were identified from coded admission diagnoses using Systematized Nomenclature of Medicine Clinical Terms (SNOMED-CT) terminology.^13^

The primary outcome was mortality in the ICU. Secondary outcomes included risk-adjusted ICU mortality, clinical management features, organ support levels (for mechanical ventilation, cardiovascular and renal support), duration of mechanical ventilation and length of stay in ICU.

### Statistical analysis

Descriptive analyses were performed to summarise the patient demographic, clinical and outcome characteristics, stratified by obstetric status. Categorical variables were presented as frequencies and percentages, and continuous variables were summarised as either mean (standard deviation) or median (interquartile range), depending upon their distribution. Due to the large imbalance in the obstetric versus non-obstetric cohort group sizes, formal hypothesis testing (e.g., chi-square tests) was not performed for group comparison, as such tests are highly sensitive to large sample sizes and may yield statistically significant results even for negligible differences. Instead, we reported absolute standardised differences (ASDs) to quantify the magnitude of imbalance between groups, with an ASD ≥ 0.10 considered to indicate a potentially meaningful difference. ASDs were calculated as standardised differences in proportions for binary variables and standardised differences for continuous variables. For variables with missing data, the number of available observations (n) was reported accordingly.

Within the obstetric cohort, additional subgroup analyses were performed for enhanced profiling of this target population. Patients were stratified by surgical status (operative versus non-operative) and by type of hospital (public versus private). Demographic, clinical and outcome characteristics of these subgroups were compared using either the chi-square test for categorical variables or the Mann–Whitney U test for continuous variables, as appropriate. No formal adjustment for multiple comparisons was applied, as these analyses were predefined descriptive subgroup comparisons rather than confirmatory hypothesis-testing analyses. To complement the reported chi-square and Mann–Whitney U test p-values, we additionally included absolute standardised differences (ASDs) to quantify the magnitude of group differences independent of sample size.

Severity-adjusted mortality was assessed using the standardised mortality ratio (SMR), in which individual predicted probabilities of death were calculated using the APACHE II model. Expected deaths were calculated by summing the individual predicted probabilities, and the SMR was defined as the ratio of observed to expected deaths (with 95% confidence intervals estimated assuming a Poisson distribution). SMRs were calculated separately for private and public ICUs and interpreted descriptively, as formal statistical comparison was limited by the small number of events. Statistical significance was set at p < 0.05 (two-tailed). All analyses were performed using R (version: 2022.12.0).

### Ethics statement

The study received approval from the institutional ethics committee of All India Institute of Medical Sciences, Bhubaneswar (T-IM/NF/Anesth12/119) in October 2024.

### Role of the funding source

The funders had no role in study design, data collection, data analysis, data interpretation, or writing of the report.

## Results

**Fig. 1:**
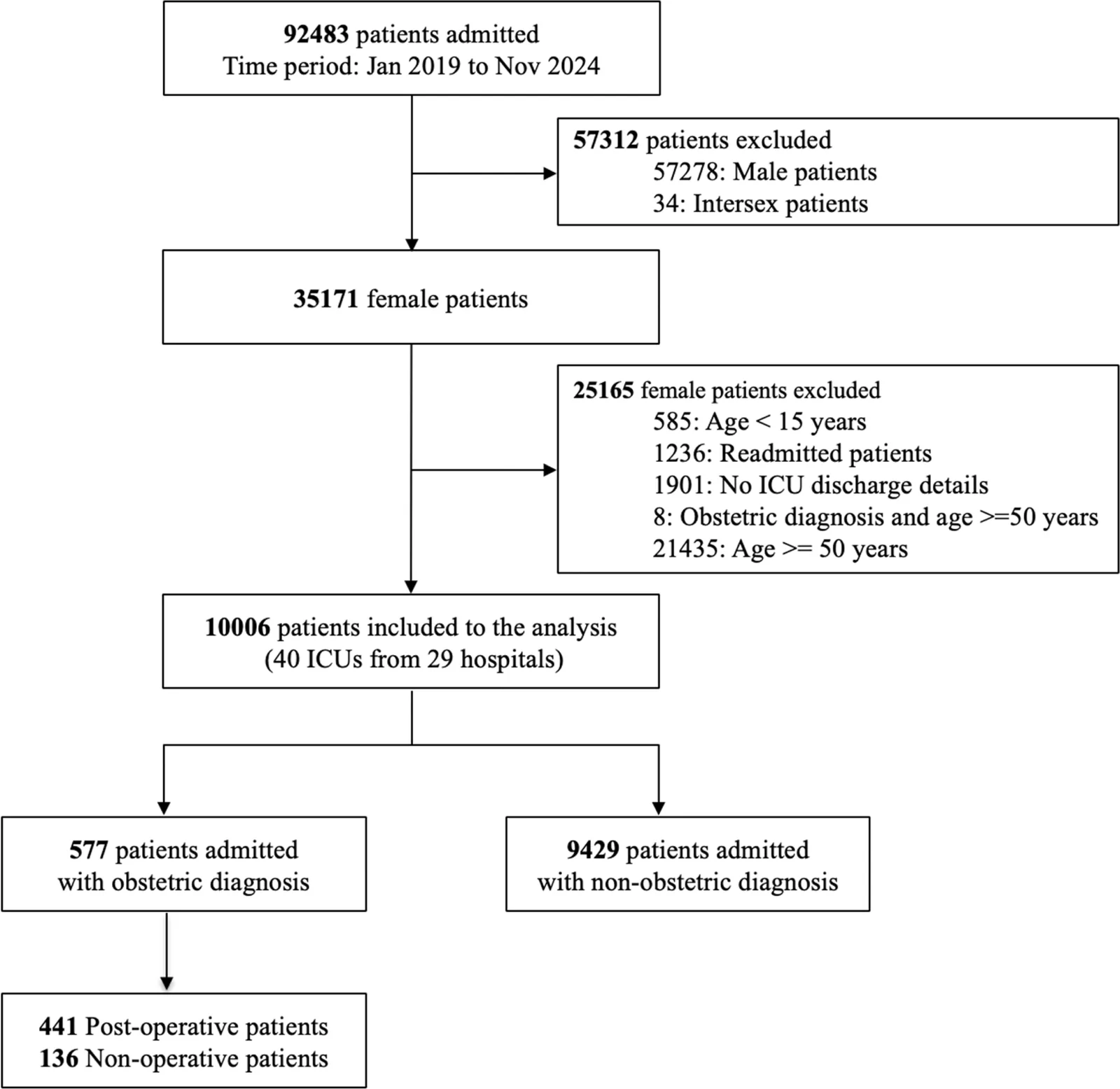
Flow diagram of the study patient cohort.

Between January 2019 and November 2024, a total of 10,006 women aged 15–49 years were admitted to the IRIS ICUs, accounting for 10.8% of all ICU admissions across 40 centres (Fig. 1). Of these, 577 (5.8%) were obstetric patients, accounting for 0.62% of the overall registry ICU admissions. The remaining 9429 (94.2%) were non-obstetric ICU admissions. The number of obstetric cases recorded in the registry progressively increased over the study period, from 740 in 2019 to 2573 in 2024, corresponding with an expansion in the number of contributing ICUs. However, the yearly proportion of admitted obstetric patients remained stable over the study period–between 5 and 7% (eTable 1 and Fig. 1). A total of 31 private ICUs and 9 public ICUs participated in the study with an heterogeneous number of obstetric ICU admissions (eTable 2). Organizational characteristics of participating units are detailed in eTable 3 and missing data variables in eTable 4.

**Fig. 2:**
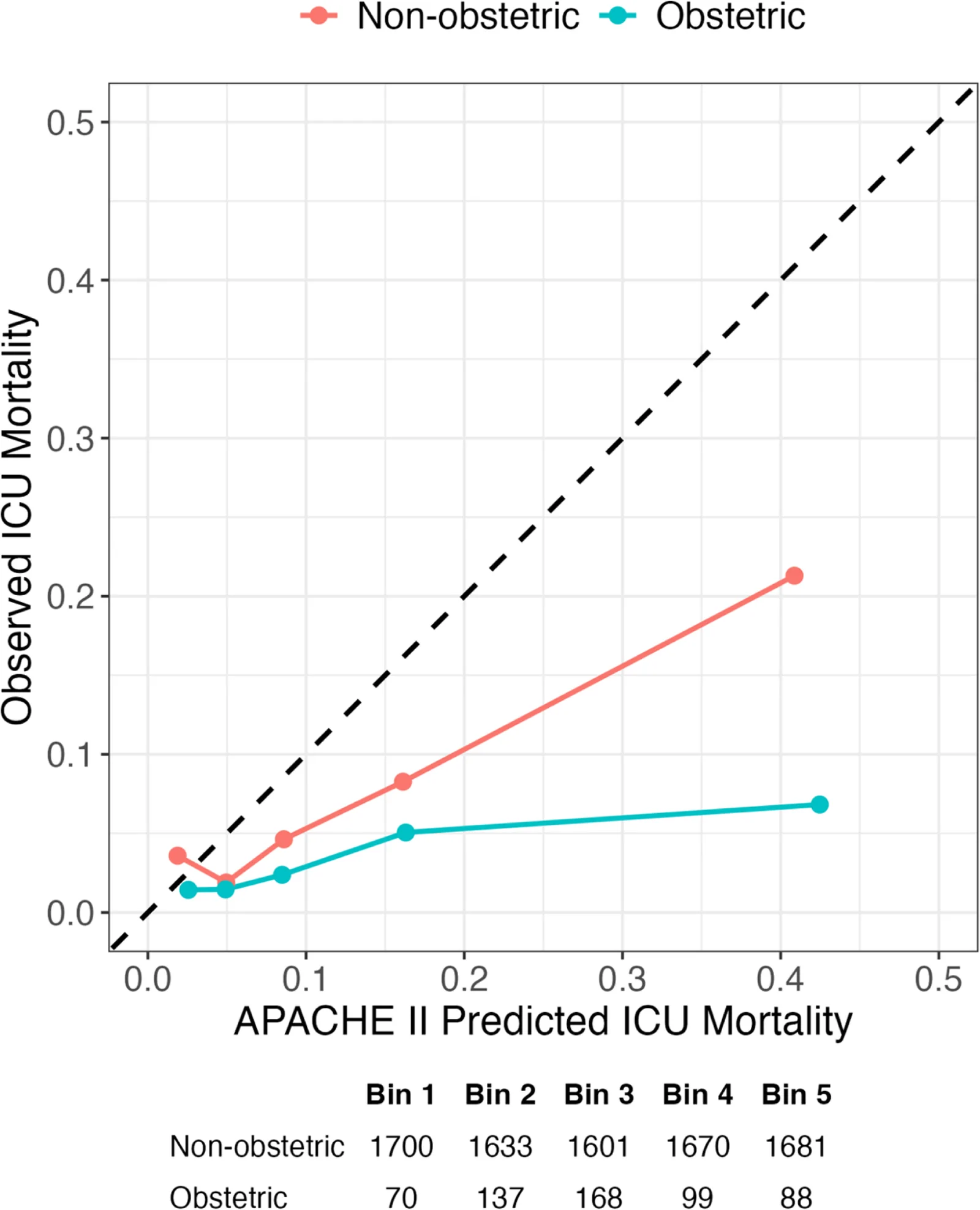
Calibration of APACHE II–predicted and observed ICU mortality stratified by obstetric status. *(Calibration was assessed using grouped predicted risk strata with observed mortality proportions).* Observed ICU mortality is plotted against APACHE II–predicted mortality for obstetric and non-obstetric women aged 15–49 years admitted to participating ICUs. The dashed diagonal line represents perfect calibration. APACHE II systematically over-predicted mortality in both groups, with greater miscalibration in obstetric patients, particularly at higher predicted risk levels. The sample sizes within each predicted risk stratum appear below the calibration plot to improve interpretation of the calibration estimates across risk groups.

As compared to their non-obstetric counterparts, obstetric patients were younger, with fewer comorbidities, and were more frequently admitted after a surgical procedure (Table 1). The median severity of illness was similar between obstetric patients and non-obstetric patients, leading to comparable predicted mortality. Organ support measures were similar between groups, with one out of four patients being intubated and invasively ventilated. One out of five patients received vasoactive drugs while RRT was rarely used, especially in the obstetric group. While obstetric patients were frequently admitted to the ICU for post-caesarean care (63%) and pre-eclampsia/eclampsia, sepsis was the leading cause of admission in the non-obstetric cohort–albeit only found in 7% of patients (eTable 5). ICU length of stay for both groups was short, with a median of 3 days. Obstetric patients had a lower crude ICU mortality (3.3%) compared to non-obstetric patients (7.9%, ASD 0.2) with APACHE II–predicted mortality consistently overestimating observed mortality (Fig. 2). Obstetric patients exhibited a biphasic pattern in cumulative deaths, with the bulk of deaths occurring in the first week of ICU admission, followed by a later increase in death events at the end of the second week, with all deaths occurring by day 14. In contrast, non-obstetric patients demonstrated a steady increase in cumulative mortality throughout the ICU stay with a substantial proportion of deaths occurring after weeks of ICU care (Supplementary Fig. S1). In additional exploratory multivariable analyses adjusting separately for APACHE II score and Charlson comorbidity category, and for age and Charlson comorbidity category, obstetric status remained independently associated with lower ICU mortality.

**Table 1:** Patient demographic, clinical care, and outcome characteristics stratified by obstetric status.

Characteristics	Total n = 10006	Obstetric patients n = 577 (5.8%)	Non-obstetric patients n = 9429 (94.2%)	ASD^a^
Age in years, median (Q1–Q3)	36 (28–43)	29 (25–33)	37 (28–44)	0.853
APACHE II Score, median (Q1–Q3)	11 (8–17)	10 (6–14)	11 (8–17)	0.158
Source of ICU admission	n = 8444	n = 491	n = 7953	
ED-same hospital	4137 (49.0)	111 (22.6)	4026 (50.6)	0.608
OR-same hospital	2656 (31.5)	290 (59.0)	2366 (29.8)	0.617
ICU/HDU-same hospital	179 (2.1)	14 (2.9)	165 (2.1)	0.050
Ward-same hospital	1385 (16.4)	66 (13.4)	1319 (16.6)	0.088
Other hospital	87 (1.03)	10 (1.7)	77 (1.0)	0.087
Admission type				1.06
Non-operative	6766 (67.6)	136 (23.6)	6630 (70.3)	
Post-operative	3240 (32.4)	441 (76.4)	2799 (29.7)	
Charlson comorbidity score, n (%)				
0	8965 (89.6)	561 (97.2)	8404 (89.1)	0.325
1–2	957 (9.6)	15 (2.60)	942 (10.0)	0.308
3–4	53 (0.5)	0 (0)	53 (0.6)	0.106
≥5	31 (0.3)	1 (0.2)	30 (0.3)	0.029
At least one organ support	n = 9917	n = 571	n = 9346	
Within the first 24 h, n (%)	3040 (30.7)	149 (26.1)	2891 (30.9)	0.107
During the ICU stay, n (%)	3451 (34.5)	165 (28.6)	3286 (34.8)	0.134
Invasive mechanical ventilation	n = 9917	n = 571	n = 9346	
Within the first 24 h, n (%)	2238 (22.6)	112 (19.6)	2126 (22.7)	0.076
During the ICU stay, n (%)	2591 (25.9)	124 (21.5)	2467 (26.2)	0.110
Non-invasive ventilation support	n = 9917	n = 571	n = 9346	
Within the first 24 h, n (%)	403 (4.1)	19 (3.3)	384 (4.1)	0.041
During the ICU stay, n (%)	619 (6.2)	32 (5.6)	587 (6.2)	0.028
Cardiovascular support	n = 9536	n = 557	n = 8979	
Within the first 24 h, n (%)	1607 (16.9)	86 (15.4)	1521 (16.9)	0.040
During the ICU stay, n (%)	1826 (18.2)	98 (17.0)	1728 (18.3)	0.035
Renal replacement therapy	n = 8926	n = 522	n = 8404	
Within the first 24 h, n (%)	261 (2.9)	6 (1.2)	255 (3.0)	0.132
During the ICU stay, n (%)	386 (3.9)	19 (3.3)	367 (3.9)	0.032
APACHE II Predicted risk of death, median (Q1–Q3)	0.08 (0.04–0.18)	0.08 (0.05–0.15)	0.08 (0.04–0.18)	0.002
ICU mortality, n (%)	768 (7.7)	19 (3.3)	749 (7.9)	0.203
SMR with 95% CI	0.59 (0.55, 0.64)	0.23 (0.13,0.37)	0.54 (0.50,0.59)	–
ICU length of stay in days, median (Q1–Q3)	3 (2–5)	3 (2–4)	3 (2–5)	<0.001

Data is reported as median (inter-quartile range) or number (percentage).

a Absolute standardised difference; an ASD of >0.1 may flag a meaningful imbalance between groups. “During ICU stay” refers to receipt of the specified organ support at any time point during the ICU admission.

Among the obstetric patients, the majority of patients were admitted from the emergency department or operating theatres of the same hospital (Table 2). The APACHE II score was significantly higher in patients with a medical admission, reflected in a significantly longer ICU length of stay (median 3 days [IQR 2–4] versus 2 days [IQR 2–3]; p-value = 0.001), albeit with lower overall exposure to cardiovascular support and invasive ventilation. ICU mortality was relatively low and did not differ significantly between groups (3.2% versus 3.4%; p = 0.78).

**Table 2:** Clinical characteristics of non-operative versus operative obstetric patients in the IRIS cohort.

Characteristics	Non-operative n = 136 (23.6%)	Operative n = 441 (76.4%)	p-value	ASD
Age in years, median (Q1–Q3)	29 (25,32)	29 (25,33)	0.310	0.106
Source of ICU admission, n (%)	n = 121	n = 370	<0.001^a^	
ED-same hospital	79 (65.3)	32 (8.7)		1.421
Operating theatre-same hospital	4 (3.3)	286 (77.3)		2.048
ICU/HDU-same hospital	8 (6.6)	6 (1.6)		0.276
Ward-same hospital	30 (24.8)	36 (9.7)		0.420
Other hospital	–	10 (2.7)		0.235
Charlson comorbidity score, n (%)			0.222	
0	131 (96.3)	430 (97.5)		0.073
1–2	4 (2.9)	11 (2.5)		0.028
>3	1 (0.7)	0 (0)		0.121
Processes of care				
Invasive ventilation support	n = 135	n = 436	0.083	
Within the first 24 h of admission, n (%)	19 (14.1)	93 (21.3)		0.188
During the ICU stay, n (%)	20 (14.7)	104 (23.6)	0.037^a^	0.222
Non-invasive ventilation support	n = 135	n = 436		
First 24 h of admission, n (%)	4 (3.0)	15 (3.4)	0.984	0.026
During the ICU stay, n (%)	5 (3.7)	27 (6.1)	0.381	0.108
Cardiovascular support	n = 132	n = 425		
First 24 h of admission, n (%)	13 (9.9)	73 (17.2)	0.116	0.211
During the ICU stay, n (%)	18 (13.2)	80 (18.1)	0.229	0.138
Renal replacement therapy	n = 126	n = 396		
First 24 h of admission, n (%)	3 (2.4)	3 (0.8)	0.164	0.129
During the ICU stay, n (%)	6 (4.4)	13 (3.0)	0.574	0.078
APACHE II score, median (Q1–Q3)	11 (8–15)	9 (6–13)	0.005^a^	0.212
ICU mortality, n (%)	5 (3.4)	14 (3.2)	0.784	0.027
SMR with 95% CI	0.24 (0.14,0.38)	0.25 (0.06,0.64)	0.23 (0.12,0.38)	–
ICU length of stay in days, median (Q1–Q3)	3 (2–4)	2 (2–3)	0.001^a^	0.322
ICU length of stay, n(%)			0.030^a^	
≤ 1 day	11 (8.1)	42 (9.5)		0.050
2 days	41 (30.1)	183 (41.5)		0.239
≥3 days	84 (61.8)	216 (49.0)		0.260

p value corresponds to the overall comparison across all categories.

APACHE, acute physiology and chronic evaluation; RRT (renal replacement therapy); ICU, intensive care unit; ASD, Absolute Standardised Difference.

a Statistical significance at the 5% level (p < 0.05).

A total of 157 obstetric patient episodes (27.2%) were encountered in public ICUs with the remaining patients recruited in private institutions. Of note, obstetric patients admitted to public ICUs were younger and sicker at admission (p < 0.001), and necessitated substantially higher organ support levels (Table 3). Crude ICU mortality was higher in public ICUs (6.4% versus 2.1%; p = 0.017), a difference that was lost after controlling for severity of illness, with a comparable standardized mortality rate which reflects the ratio between observed and expected ICU deaths.

**Table 3:** Clinical characteristics of obstetric patients admitted to public versus private ICUs.

Characteristics	Obstetric patients admitted to public ICUs (n = 157)	Obstetric patients admitted to private ICUs (n = 420)	p-value	ASD
Age in years, median (Q1–Q3)	27 (24–32)	30 (26–33)	<0.001^b^	0.328
Admission type, n (%)			0.060	
Non-operative	28 (17.8)	108 (25.7)		0.190
Post-operative	129 (82.2)	312 (74.3)		0.190
APACHE II Score, median (Q1–Q3)	12 (8–19)	8 (6–12)	<0.001^b^	
Invasive ventilation support during the ICU stay, n (%)	71 (45.2)	53 (12.6)	<0.001^b^	0.765
Cardiovascular support during the ICU stay, n (%)	64 (40.8)	34 (8.1)	<0.001^b^	0.821
RRT during ICU stay, n (%)	8 (5.1)	11 (2.6)	0.221	0.132
ICU mortality, n (%)	10 (6.4)	9 (2.1)	0.017^b^	0.219
APACHE II Predicted risk of death			<0.001^b^	–
Median (Q1–Q3)	0.12 (0.07–0.25)	0.06 (0.04–0.12)		
Mean (standard deviation)	0.19 (0.16)	0.11 (0.13)	a	0.590
SMR with 95% CI	0.34 (0.16, 0.63)	0.19 (0.08, 0.36)	0.201	–
ICU length of stay in days, median (Q1–Q3)	3 (2–4)	3 (2–3)	0.024^b^	0.106

APACHE, acute physiology and chronic evaluation; RRT, renal replacement therapy; SMR, standardized mortality rate; ICU, intensive care unit.

a p-value for the mean APACHE II predicted risk of death not computed due to skewed distribution, yet values are provided as this is utilized to compute the SMR.

b Statistical significance at the 5% level (p < 0.05).

## Discussion

In this multicentre registry-based cohort in India, critically ill obstetric patients differed systematically from non-obstetric women of reproductive age in terms of case-mix, admission pathways, ICU trajectories, and outcomes, despite having broadly similar acuity of illness on admission and organ support requirements. APACHE II consistently over-predicted mortality in obstetric patients when compared with observed outcomes, reflecting physiological miscalibration of a severity score not designed for pregnancy-related adaptations. Obstetric admissions with a medical condition were more severely ill on admission and required longer stays in ICU, albeit with lower organ support and comparable mortality as compared to surgical obstetric patients. Importantly, public ICUs faced patients with higher illness severity, distinct referral pathways, and higher crude mortality—which did not translate in worse ICU performance, as evidenced by comparable severity-adjusted mortality.

The proportion of obstetric admissions to the ICU in our cohort (0·6%) was similar to reports from high-income settings such as Australia (1·3%), Japan (0·4%), and Portugal (0·9%),^14^, ^15^, ^16^ and lower than those reported from Ghana (12·8%), Sierra Leone (11%),^4^^,^^17^ and other LMICs, demonstrating geographic and socioeconomic differences in healthcare availability and utilisation. Within the obstetric cohort, severity of illness, ICU trajectory, and resource utilisation differed significantly between operative and emergency–medical admissions. Higher severity scores and longer ICU stays among non-operative obstetric cases suggest that this subgroup represents a more acutely ill population, often admitted with medical or obstetric emergencies rather than for elective or perioperative care. The slightly higher rates of ventilation and vasopressors in the surgical group may be explained by this group of patients often arriving from the operating room for weaning purposes, which is accomplished in a relatively short period of time.

In our cohort three out of 100 parturients admitted to ICU died as a consequence of the critical illness arising from direct or indirect obstetric complications. In this context, the lower mortality observed in our cohort compared with other LMIC reports represents an important finding that aligns these Indian ICUs more towards high-income setting ICU outcomes.^4^ Between 2000 and 2023, the global maternal mortality ratio declined by approximately 40%.^2^ In India, similar to Brazil and South Africa,^18^^,^^19^ the National Health Mission has strengthened public-sector maternal health services, with antenatal care coverage and institutional deliveries increasing from 57% to 94% and from 34% to 90%, respectively. Over this period, the maternal mortality rate declined from 212 to 97 deaths per 100,000 live births, alongside mandatory reporting of maternal deaths and severe maternal morbidity. These measures likely lowered the threshold for escalation of care and ICU referral, including for relatively less severe conditions,^1^^,^^20^ providing a plausible explanation for the lower severity of illness and mortality observed in this analysis. An additional consideration relates to the design of the registry itself. As an opt-in registry, IRIS may over-represent ICUs with established data collection and quality-improvement practices. However, participating units included both public and private ICUs across diverse settings, and the observed differences in outcomes were largely explained by differences in severity of illness at admission rather than by ICU type itself.^21^^,^^22^

Consistent with prior reports, APACHE II over-predicted mortality in obstetric cohorts, especially evident in our cohort in direct comparison with non-obstetric women of the same age.^16^ This miscalibration likely reflects the application of physiological thresholds derived from non-pregnant populations to obstetric patients, in whom normal pregnancy-related changes in cardiovascular, respiratory, and laboratory parameters may inflate severity scores without corresponding increases in mortality risk.^23^

Although fewer public ICUs were represented in the registry compared with private ICUs, the level of some staffing metrics was broadly comparable. Critical illness in obstetrics occurs either due to major obstetric complications or following delays in access to care, as described in the three-delays model, encompassing delays in seeking care, reaching an appropriate health facility, and receiving adequate treatment once care is accessed.^24^ The notable greater severity of illness at admission observed in public ICUs suggests gaps in prehospital care or delayed referral, particularly for complicated and marginalised patients, who may benefit from targeted strengthening of obstetric emergency and critical care pathways and more timely referral.^25^ While this pattern is not unique to the Indian setting or LMICs,^26^ improving equity and value across costs and outcomes remains a fundamental priority in maternity care, including maternal critical care. ICU registries enable these upstream effects to be examined once adjusted for severity. We therefore reported both observed mortality and standardized mortality ratios (SMRs) to provide complementary perspectives on outcomes across public and private ICUs. The SMR is derived from APACHE II predicted mortality and therefore partially adjusts for differences in age, physiological severity, and chronic health status between groups. However, residual confounding related to referral pathways, delays in presentation, resource availability, and case-mix differences may persist despite risk adjustment. Given the descriptive objectives of this registry-based study and the relatively small number of deaths within some subgroups, additional multivariable modelling was not performed. Our findings illustrate how ICU registries in LMICs can function as learning health systems, complementing maternal health surveillance through severity-adjusted comparisons across patient groups and health-system contexts.^27^

At the lower end of the severity spectrum, the predominance of short-stay postoperative admissions may support the fostering of obstetric high-dependency or step-down units to optimise ICU bed utilisation without compromising patient safety.^28^ At the same time, the comparable acuity of illness and need for organ support between obstetric and non-obstetric women emphasise the importance of strengthening early recognition, timely referral, and escalation pathways for critically ill obstetric patients. Through low cost and near real time data collection ICU registries may contribute to identify delays in care, inform quality improvement initiatives and correct bed allocation scaled by patient severity.^29^

This study has several strengths. Firstly, The multicentre design with inclusion of both public and private ICUs across diverse settings lends robustness to findings. The use of a cloud-based standardised registry using a common data model, allows for comparisons between obstetric and non-obstetric critical illness. We could not avoid inherent limitations of registry-based designs such as variability in diagnostic coding practices (mitigated by standardized validated nomenclature such as SNOMED-CT in the registry platform), and lack of long-term outcomes (e.g., mortality after ICU discharge, neurological or long term outcomes). The absence of cause-specific mortality data must also be acknowledged. Although missing data are an inherent limitation of registry-based studies, completeness of APACHE-II component data exceeded 88% and organ support variables showed 90–100% availability across the cohort. We acknowledge that the predominance of private centres has introduced a selection bias, potentially leading to underestimation of mortality and limiting generalisability for rural and lower-resource settings. Although no statistically significant difference in SMR was observed between public and private ICUs, the subgroup analysis may have been underpowered due to the relatively small number of public-sector centres and events. This underscores the need to expand critical care registries to district hospitals and under-represented public-sector ICUs.

In conclusion, a critically ill obstetric patient cohort can present a distinct ICU phenotype compared to non-obstetric women. While three out of one hundred patients still die in Indian ICUs, severity of illness scores such as APACHE II may overestimate mortality highlighting the need for model recalibration to better guide triage and resource allocation. Participants in this study were shown to have higher severity, longer ICU stays but without an extensive organ support burden as compared to surgical patients. Observed differences between public versus private healthcare systems primarily reflect upstream health system and socioeconomic factors influencing access to timely critical care, underscoring the importance of strengthening pre-ICU referral pathways to improve equity of care for obstetric patients in LMICs. Future research should prioritise the identification of modifiable predictors of maternal morbidity and mortality, long-term maternal outcomes, and development context-appropriate models for high-dependency care and early intervention to further improve outcomes for critically ill obstetric patients.

## Contributors

ST, NS, BKTV and LP conceptualised the study. ST, NS, DGD and LP developed the methodology. ST conducted the investigation. ST and DGD curated the data. DGD validated the data. ST and DGD performed the formal analyses. ST supervised and administered the project. DGD prepared the visualisations. ST drafted the original manuscript. ST, DGD, BKTV, NS, SS, RH, AB and LP reviewed and edited the manuscript. BKTV, NS, SS, RH, AB and LP critically revised the manuscript for important intellectual content. LP provided senior authorship oversight. ST was the corresponding author. ST, LP and DGD and verified the underlying data. All authors had full access to the reported data and approved the final manuscript All authors approved the final version of the manuscript and accept responsibility for the decision to submit it for publication.

## Data sharing statement

De-identified participant data underlying the findings of this study may be made available upon reasonable request to the corresponding author, subject to registry governance and institutional approvals.

## Declaration of AI use

During manuscript preparation the author(s) used ChatGPT (OpenAI) to assist with language refinement and structural suggestions. No data were generated by the tool, and all conceptual content, interpretation, analysis and conclusions are original to the authors. After reviewing and editing the tool’s contributions, the authors take full responsibility for the content of the published article.

## Declaration of interests

We declare no competing interests.

## Appendix A Supplementary data

eTables 1–5

Full list of collaborators

## References

[1] Targets of sustainable development goal 3. https://www.who.int/europe/about-us/our-work/sustainable-development-goals/targets-of-sustainable-development-goal-3.

[2] Say L., Chou D., Gemmill A. (2014). Global causes of maternal death: a WHO systematic analysis. Lancet Glob Health.

[3] Marotta C., Pisani L., Di Gennaro F. (2020). Epidemiology, outcomes, and risk factors for mortality in critically ill women admitted to an obstetric high-dependency unit in Sierra Leone. Am J Trop Med Hyg.

[4] Tripathy S., Singh N., Panda A. (2024). Critical care admissions and outcomes in pregnant and postpartum women: a systematic review. Intensive Care Med.

[5] Raina N., Khanna R., Gupta S., Jayathilaka C.A., Mehta R., Behera S. (2023). Progress in achieving SDG targets for mortality reduction among mothers, newborns, and children in the WHO South-East Asia Region. Lancet Reg Health Southeast Asia.

[6] Abie A., Mehari M.G., Dagnew T.E. (2025). Obstetric admission and maternal mortality in the intensive care unit in Africa: a systematic review and meta-analysis. PLoS One.

[7] Rojas-Suarez J., Paruk F. (2024). Maternal high-care and intensive care units in low- and middle-income countries. Best Pract Res Clin Obstet Gynaecol.

[8] IRIS ICU Registry – collaboration between NICST & CCCG. https://irisicuregistry.org/.

[9] Pisani L., Rashan T., Shamal M. (2021). Performance evaluation of a multinational data platform for critical care in Asia. Wellcome Open Res.

[10] Tirupakuzhi Vijayaraghavan B.K., Adhikari N.K.J., Arali R. (2020). Implementing an intensive care registry in India: preliminary results of the case-mix program and an opportunity for quality improvement and research. Wellcome Open Res.

[11] STROBE statement-checklist of items that should be included in reports of cohort studies. http://www.epidem.com/.

[12] Beane A., Dondorp A.M., Taqi A. (2020). Establishing a critical care network in Asia to improve care for critically ill patients in low- and middle-income countries. Crit Care.

[13] Home | SNOMED International. https://www.snomed.org/.

[14] Maiden M.J., Finnis M.E., Duke G.J. (2020). Obstetric admissions to intensive care units in Australia and New Zealand: a registry-based cohort study. BJOG.

[15] Naruse S., Nakajima M., Aoki Y. (2025). Clinical features and outcomes of peripartum obstetric patients admitted to the intensive care unit: a nationwide inpatient database in Japan. Crit Care.

[16] Ryan H.M., Sharma S., Magee L.A. (2016). The usefulness of the APACHE II score in obstetric critical care: a structured review. J Obstet Gynaecol Can.

[17] Pirzada S., Boswell K., Yang J. (2024). Clinical characteristics and outcomes of obstetric patients transferred directly to intensive care units. Acute Critical Care.

[18] Chopra M., Daviaud E., Pattinson R., Fonn S., Lawn J.E. (2009). Saving the lives of South Africa’s mothers, babies, and children: can the health system deliver?. Lancet.

[19] Albuquerque P.C., Felipe L.L., Lopes J.F., de Tassinari W.S., Zicker F., de Fonseca B.P. (2025). Geographic accessibility to hospital childbirths in Brazil (2010–2011 and 2018–2019): a cross-sectional study. Lancet Reg Health Am.

[20] (2013). Guidelines for maternal death surveillance & reSponSe. https://www.who.int/publications/i/item/9789241506083.

[21] Ohbe H., Kudo D., Kobayashi N. (2025). Timing of onset of persistent critical illness in Japan: a nationwide registry study. Lancet Reg Health West Pac.

[22] Irie H., Okamoto H., Uchino S. (2020). The Japanese intensive care patient database (JIPAD): a national intensive care unit registry in Japan. J Crit Care.

[23] Chimwaza Y., Hunt A., Oliveira-Ciabati L. (2025). Early warning systems for identifying severe maternal outcomes: findings from the WHO global maternal sepsis study. EClinicalMedicine.

[24] Three delays inducing maternal morbidity and mortality–public health notes. https://publichealthnotes.com/three-delays-for-maternal-morbidity-and-mortality/.

[25] Chauke L. (2025). Improving access to emergency obstetric care in low- and middle-income countries. Best Pract Res Clin Obstet Gynaecol.

[26] Callander E.J., Enticott J., Mol B.W. (2025). Maternal and neonatal outcomes and health system costs in standard public maternity care compared to private obstetric-led care: a population-level matched cohort study. BJOG.

[27] Salluh J.I.F., Quintairos A., Dongelmans D.A. (2024). National ICU registries as enablers of clinical research and quality improvement. Crit Care Med.

[28] Gu N., Zheng Y., Dai Y. (2022). Severe maternal morbidity: admission shift from intensive care unit to obstetric high-dependency unit. BMC Pregnancy Childbirth.

[29] Beane A., Salluh J.I.F., Haniffa R. (2021). What intensive care registries can teach us about outcomes. Curr Opin Crit Care.

