# Supplementary material for: Critical care utilisation, and outcomes of critically ill obstetric patients in India: a multicentre ICU registry study (2019–2024)
Source: Lancet Reg Health Southeast Asia. 2026 Aug 26;52:100845. doi: 10.1016/j.lansea.2026.100845 (PMC13544171; doi:10.1016/j.lansea.2026.100845)
Supplement: Supplementary file 1 [file mmc1.docx]

**SUPPLEMENTARY MATERIAL**

**Case-mix, critical care utilisation, and outcomes of critically ill obstetric patients in India: a multicentre ICU registry study**

[eTable 1: Number of patients admitted to ICUs over the years.	2](#_Toc220767586)

[eTable 2: Number of patients admitted to ICUs	3](#_Toc220767587)

[eTable 3. Main organizational characteristics of participating ICUs stratified by ICU status.	4](#_Toc220767588)

[eTable 4. Availability of core variables used in the analysis (N = 10,006)	5](#_Toc220767589)

[eTable 5. Reason for ICU admission as per the APACHE IV coding.	6](#_Toc220767590)

# eTable 1: Number of patients admitted to ICUs over the years.

Although the absolute number of obstetric ICU admissions increased over time in parallel with expansion of participating ICUs, the proportion of obstetric admissions remained relatively stable, ranging between approximately 5% and 7% across study years. This suggests that the observed increase in case numbers reflects registry growth rather than a temporal increase in obstetric critical illness.

| Year | **2019** | **2020** | **2021** | **2022** | **2023** | **2024 (Jan-Nov)** | **Total** |
| --- | --- | --- | --- | --- | --- | --- | --- |
| All ICU admissions* | 6703 | 7729 | 12546 | 19334 | 21097 | 25074 | 92483 |
| Eligible ICU admissions^†^ | 740 | 814 | 1404 | 2028 | 2447 | 2573 | 10006 |
| Obstetric ICU admissions^+^ | 34 (4.6%) | 54  (6.6%) | 69  (4.9%) | 116 (5.7%) | 134  (5.5%) | 170  (6.6%) | 577  (5.8%) |
| Number of ICUs** | 11 | 20 | 31 | 30 | 29 | 39 | 40 |

**All ICU admissions recorded in the registry for the given year*

*^†^Admissions meeting the study’s inclusion/exclusion criteria (study cohort)*

*^+^n (%) within eligible ICU admissions for that year*

*** only ICU reporting at least one eligible patient to the study cohort*

# eTable 2: Number of patients admitted to ICUs

| **ICU ID** | **ICU type** | **Number of women aged 15-49 yrs.** | **Number of obstetric patients** | **Funding model** |
| --- | --- | --- | --- | --- |
| 1 | Mixed | 80 | 1 | Private |
| 2 | Special | 11 | NA | Public |
| 3 | Special | 44 | 1 | Public |
| 4 | Mixed | 16 | NA | Public |
| 5 | Special | 11 | NA | Public |
| 6 | Special | 98 | 2 | Public |
| 7 | Mixed | 1148 | 140 | Public |
| 8 | Special | 428 | 1 | Public |
| 9 | Medical | 4 | 1 | Private |
| 10 | Medical | 12 | 1 | Private |
| 11 | Mixed | 241 | NA | Private |
| 12 | Special | 153 | NA | Private |
| 13 | Mixed | 362 | 2 | Private |
| 14 | Mixed | 461 | 50 | Private |
| 15 | Mixed | 805 | 21 | Private |
| 16 | Mixed | 500 | NA | Private |
| 17 | Mixed | 897 | 32 | Private |
| 18 | Mixed | 826 | 34 | Private |
| 19 | Mixed | 137 | 20 | Private |
| 20 | Special | 508 | 27 | Private |
| 21 | Surgical | 21 | NA | Private |
| 22 | Medical | 30 | 1 | Private |
| 23 | Mixed | 47 | 1 | Private |
| 24 | Medical | 141 | 10 | Private |
| 25 | Medical | 24 | 2 | Private |
| 26 | Mixed | 187 | 12 | Public |
| 27 | Mixed | 31 | 4 | Private |
| 28 | Mixed | 16 | 4 | Private |
| 29 | Mixed | 47 | 1 | Private |
| 30 | Medical | 367 | 27 | Private |
| 31 | Mixed | 323 | 71 | Private |
| 32 | Medical | 11 | NA | Private |
| 33 | Mixed | 165 | 4 | Private |
| 34 | Medical | 55 | 1 | Private |
| 35 | Surgical | 20 | NA | Private |
| 36 | Medical | 299 | 28 | Private |
| 37 | Medical | 733 | 70 | Private |
| 38 | Surgical | 63 | 1 | Private |
| 39 | Mixed | 444 | 1 | Public |
| 40 | Mixed | 240 | 6 | Private |

# eTable 3. Main organizational characteristics of participating ICUs stratified by ICU status.

| **Domain** | **All ICUs**  ***(N=40)*** | **Public**  ***(N=9)*** | **Private**  ***(N=31)*** |
| --- | --- | --- | --- |
| **Number Of Beds** | 667 | 125 | 542 |
| **Presence of an intensivist, n (%)** | 28(70%) | 6 (67%) | 22 (70%) |
| **ICU Certified in-charge nurse, n (%)** | 17 (43%) | 3 (43%) | 14 (45%) |
| **Nurse to Patient Ratio, median (IQR) n= Number of ICUs contributing data** | 1:2  n=23 | 1:2  N=6 | 1:2  N=17 |

# eTable 4. Availability of core variables used in the analysis (N = 10,006)

| **Variable** | **Availability, n (%)** |
| --- | --- |
| Age in years | 10006 (100.0) |
| APACHE II Score | 9832 (98.3) |
| Source of ICU admission | 8444 (84.4) |
| Admission type | 10006 (100.0) |
| Charlson Comorbidity Score | 10006 (100.0) |
| Invasive mechanical ventilation at admission | 9917 (99.1) |
| Non-invasive ventilation support at admission | 9917 (99.1) |
| Cardiovascular support at admission | 9536 (95.3) |
| Renal Replacement Therapy at admission | 8926 (89.2) |
| APACHE II predicted risk of death | 8847 (88.4) |
| ICU mortality | 10006 (100.0) |
| ICU length of stay | 10006 (100.0) |

eTable 5. Reason for ICU admission as per the APACHE IV coding. The reason for performing caesarean sections (i.e. most major direct obstetric complications) cannot be detailed by this method hence may contain cases pertaining also to other categories e.g. sepsis or severe pre-eclampsia.

| **Primary reason for ICU admission**  (APACHE IV diagnoses top 10) | **N(%)** |
| --- | --- |
| **For obstetric patients**, n(%), n = 562  Cesarean section  Pre-eclampsia/eclampsia  GI surgery, other  Ectopic pregnancy  Genitourinary medical, other  Hemorrhage, postpartum  Genitourinary surgery, other  Hypertension, uncontrolled  Sepsis, other  Seizures (primary-no structural brain disease) | 354 (63.0)  65 (11.6)  35 (6.2)  20 (3.6)  15 (2.7)  14 (2.5)  10 (1.8)  6 (1.1)  5 (0.9)  4 (0.7) |
| **For non-obstetric patients, n(%)**, n = 8333  Sepsis, other  GI surgery, other  Overdose, other toxin, poison or drug  Genitourinary medical, other  Cranioplasty and complications from previous craniotomies  Seizures (primary-no structural brain disease)  GI medical, other  CVA, cerebrovascular accident/stroke  Respiratory- medical, other  Neurologic medical, other | 587 (7.0)  375 (4.5)  371 (4.5)  355 (4.3)  310 (3.7)  288 (3.5)  245 (2.9)  215 (2.6)  201 (2.4)  171 (2.1) |

*To note that diagnostic categories are APACHE IV-based, but severity scoring is APACHE II.
