# Supplementary material for: Critical care utilisation, and outcomes of critically ill obstetric patients in India: a multicentre ICU registry study (2019–2024)
Source: Lancet Reg Health Southeast Asia. 2026 Aug 26;52:100845. doi: 10.1016/j.lansea.2026.100845 (PMC13544171; doi:10.1016/j.lansea.2026.100845)
Supplement: Supplementary file 2 [file mmc2.docx]

| **S. no.** | **Site** | **Contributors** | **Email address** |
| --- | --- | --- | --- |
| 1 | ABC Hospital, Visakhapatnam, Andhra Pradesh | Dr Kavitha Ramesh | kavita.pattipati@gmail.com |
|  | ABC Hospital, Visakhapatnam, Andhra Pradesh | Dr Saradha | saradach.22@gmail.com |
| 2 | AIIMS, Bhubaneswar, Odisha | Dr Swagata Tripathy | tripathyswagata@gmail.com |
|  | AIIMS, Bhubaneswar, Odisha | Ms Kasturi Sanyal | kasturi.iris@gmail.com |
|  | AIIMS, Bhubaneswar, Odisha | Mr Sunil | suniliris2024@gmail.com |
| 3 | AMRI Hospitals, Bhubaneswar, Odisha | Dr Samir Sahu | samirsahu_kal@yahoo.co.in |
| 4 | Amrita Institute of Medical Sciences, Kochi, Kerala | Dr Zubair Umer Mohamed | zubairumer@gmail.com |
|  | Amrita Institute of Medical Sciences, Kochi, Kerala | Ms Anna Paul | annapaul9619@gmail.com |
| 5 | Apollo Cancer Institute, Chennai | Dr Dedeepiya Devaprasad | dedeepiya@icuconsultants.com |
|  | Apollo Cancer Institute, Chennai | Dr Rohit Aravindakshan Kooloth | rohit@icuconsultants.com |
|  | Apollo Cancer Institute, Chennai | Dr Sandeep Shyamsundar | sandeep@icuconsultants.com |
|  | Apollo Cancer Institute, Chennai | Mr Vijay | vijaymph98@gmail.com |
|  | Apollo Cancer Institute, Chennai | Mr Deepak Raj | sdeepakraj1997@gmail.com |
|  | Apollo Cancer Institute, Chennai | Ms Sowmiya Panneer Selvam | sowmiyapanneer22@gmail.com |
| 6 | Apollo First Med Hospital, Chennai | Dr Meghena Mathew | meghena@icuconsultants.com |
|  | Apollo First Med Hospital, Chennai | Ms Preethika Ravichandran | preethikaravi98@gmail.com |
|  | Apollo First Med Hospital, Chennai | Mr Lakshmi Narashimhan Srinivasan | lakshminarashimhansrinivasan@gmail.com |
| 7 | Apollo Main Hospital, Chennai | Dr Bharath | bharath@icuconsultants.com |
|  | Apollo Main Hospital, Chennai | Mr Augustian James | augustian.j@gmail.com |
|  | Apollo Main Hospital, Chennai | Ms Sruthi Mano | sruthimano278@gmail.com |
|  | Apollo Main Hospital, Chennai | Mr Mithilesh Devaraj | mithilesh.cccc54@gmail.com |
| 8 | Apollo Proton Cancer Centre, Chennai | Dr Raymond Dominic Savio | raymond@icuconsultants.com |
|  | Apollo Proton Cancer Centre, Chennai | Dr Suresh Kumar Sundaramurthy | suresh@icuconsultants.com |
|  | Apollo Proton Cancer Centre, Chennai | Ms Vinisha Varadharajan | vinishavaradhan8@gmail.com |
| 9 | Apollo Speciality Hospital – OMR, Chennai | Dr Pratheema Ramachandran | pratheema@icuconsultants.com |
|  | Apollo Speciality Hospital – OMR, Chennai | Ms Swedha Vasudevan | swedhavasudevan23@gmail.com |
|  | Apollo Speciality Hospital – OMR, Chennai | Ms Priyadharshini Vasudevan | priyadharshinivasu19@gmail.com |
| 10 | Apollo Speciality Hospital, Vanagaram, Chennai | Dr Ebenezer Rabindrarajan | ebenezer@icuconsultants.com |
|  | Apollo Speciality Hospital, Vanagaram, Chennai | Dr Vanishree Chandran | vanishree@icuconsultants.com |
|  | Apollo Speciality Hospital, Vanagaram, Chennai | Ms Mohana Priya Sachidhanandam | mohanapriya20234@gmail.com |
| 11 | Bharati Hospital and Research Centre | Dr Jignesh Shah | jignesh.shah@bharatividyapeeth.edu |
|  | Bharati Hospital and Research Centre | Dr Digamber singh | digamber.singh@bharatihospital.com |
|  | Bharati Hospital and Research Centre | Mr Vikas Munde | icutrials@gmail.com |
| 12 | Dr. Kamakshi Memorial Hospital, Pallikaranai, Chennai | Dr Devachandran Jayakumar | devajayakumar1@gmail.com |
|  | Dr. Kamakshi Memorial Hospital, Pallikaranai, Chennai | Dr Dinesh | dineshprathick2@gmail.com |
|  | Dr. Kamakshi Memorial Hospital, Pallikaranai, Chennai | Dr Vignesh R | dr.r.vignesh@gmail.com |
|  | Dr. Kamakshi Memorial Hospital, Pallikaranai, Chennai | Avinash RM | avinash.rm1993@gmail.com |
|  | Dr. Kamakshi Memorial Hospital, Pallikaranai, Chennai | Ms Lavanya Elumalai | lavanyae2507@gmail.com |
|  | Dr. Kamakshi Memorial Hospital, Pallikaranai, Chennai | Dr Sucharita Yanamandra | sucharitayanamandra@outlook.com |
| 13 | Eternal Hospital, Jaipur, Rajasthan | Dr Kishore Mangal | drkishoremangal@gmail.com |
| 14 | Fortis Escorts Hospital, Jaipur, Rajasthan | Dr Kishore Mangal | drkishoremangal@gmail.com |
|  | Fortis Escorts Hospital, Jaipur, Rajasthan | Dr Vaibhav Bhargava | vaibhav.bhargava@fortishealthcare.com |
|  | Fortis Escorts Hospital, Jaipur, Rajasthan | Ms Seema Kumari Gupta | guptaseema53@gmail.com |
| 15 | IQRAA Hospital, Kozhikode, Kerala | Dr Niyaz Channanath Ashraf | niyazashraf@gmail.com |
| 16 | ISPAT General Hospital, Rourkela, Odisha | Dr Chinni Krishna Kasi | ck.kasi@gmail.com |
|  | ISPAT General Hospital, Rourkela, Odisha | Mr Durga Madhab Tripathi | tdurgamadhab@gmail.com |
| 17 | KIMS, Tiruvananthapuram, Kerala | Dr Deepak | deepak.v@kimsglobal.com |
|  | KIMS, Tiruvananthapuram, Kerala | Ms Sree lakshmi | clinicalresearch@kimshealth.org |
| 18 | Manipal Hospitals | Dr Rajesh Mohan Shetty | rajeshshetty@manipalhospitals.com |
|  | Manipal Hospitals | Dr Shivangi Mishra | shivangi.mishra@manipalhospitals.com |
|  | Manipal Hospitals | Ms Sangeetha G paulraj | sangeethagilbertpaulraj@gmail.com |
|  | Manipal Hospitals | Ms Sheethal | ksheeth@gmail.com |
| 19 | Medanta The Medicity, Gurugram, Haryana | Dr Yatin Mehta | yatin.mehta@medanta.org |
|  | Medanta The Medicity, Gurugram, Haryana | Mr Joby V George | joby.george@medanta.org |
|  | Medanta The Medicity, Gurugram, Haryana | Mr Shaheen Sheikh | shaheen.sheikh@medanta.org |
| 20 | Medicover Hospital, Navi Mumbai | Dr Aditi jain | DrAditiJain@gmail.com |
|  | Medicover Hospital, Navi Mumbai | Mr Shubam Dhokiya | shubhamdhokiya7530@gmail.com |
| 21 | Mehta Hospital, Chennai | Dr Jaganathan Selvanayagam | jaganintensivist@gmail.com |
|  | Mehta Hospital, Chennai | Mr Thirumalai Sampath | thirumalai031999@gmail.com |
| 22 | Nanjappa Multi-Speciality Hospital, Karnataka | Dr Rakesh Lakshmappa | mlrakesh4239@gmail.com |
| 23 | Pushpagiri Medical College Hospital, Tiruvalla, Kerala | Dr Mathew Pulicken | pulicken@yahoo.com |
| 24 | Sir H N Reliance Foundation Hospital, Mumbai, Maharashtra | Dr Darshana rathod | rathoddarshana10@gmail.com |
| 25 | SMBT, Nasik , Maharashtra | Dr Ruchira khasne | drruchirakhasne@gmail.com |
|  | SMBT, Nasik , Maharashtra | Mr Namesh Ananp | nameshanap93@gmail.com |
| 26 | Symbiosis University Hospital and research centre, Pune | Dr Urvi Shukla | urvi.shukla@suhrc.siu.edu.in |
|  | Symbiosis University Hospital and research centre, Pune | Dr Amol Bali | amol.bali@suhrc.siu.edu.in |
|  | Symbiosis University Hospital and research centre, Pune | Ms Chinmayee A Bhise | researchcoordinator@suhrc.siu.edu.in |
| 27 | Tata Memorial Hospital, Mumbai | Dr Atul Kulkarni | kaivalyaak@yahoo.co.in |
|  | Tata Memorial Hospital, Mumbai | Dr Anjana Shrivastava | anjanashri@gmail.com |
|  | Tata Memorial Hospital, Mumbai | Ms Nikita Kulaye | nkalan8@gmail.com |
|  | Tata Memorial Hospital, Mumbai | Mr Karuna Panahle | karunapanahle@gmail.com |
| 28 | Vijaya Medical and Educational Trust, Chennai | Dr Balaji Venkatachalam | balaji@icuconsultants.com |
|  | Vijaya Medical and Educational Trust, Chennai | Dr Pavan kumar Vecham | pavan@icuconsultants.com |
|  | Vijaya Medical and Educational Trust, Chennai | Ms Mariswari Raja | mariswariraja2477@gmail.com |
