# Supplementary material for: Critical care utilisation, and outcomes of critically ill obstetric patients in India: a multicentre ICU registry study (2019–2024)
Source: Lancet Reg Health Southeast Asia. 2026 Aug 26;52:100845. doi: 10.1016/j.lansea.2026.100845 (PMC13544171; doi:10.1016/j.lansea.2026.100845)
Supplement: Supplementary file 3 [file mmc3.docx]

Supplement Table 1

Supplementary Table: Multivariable logistic regression analyses for ICU mortality

|  | Adjusted OR (95% CI) | p-value |
| --- | --- | --- |
| Model 1: Adjusted for APACHE II score and Charlson category |  |  |
| Obstetric vs non-obstetric | 0.47(0.28–0.74) | 0.002 |
| APACHE II Score | 1.13(1.12–1.14) | <0.001 |
| Charlson category 0 | 0.29(0.13–0.70) | 0.004 |
| Charlson category 1-2 | 0.19(0.08–0.47) | <0.001 |
| Charlson category 3-4 | 0.09(0.02–0.32) | <0.001 |
| Charlson category ≥5 | Ref | - |
| Model 2: Adjusted for age and Charlson comorbidity category |  |  |
| Obstetric vs non-obstetric | 0.42(0.25–0.65) | <0.001 |
| Admission age | 1.01(1.00–1.02) | 0.024 |
| Charlson category 0 | 0.18(0.09–0.40) | <0.001 |
| Charlson category 1-2 | 0.17(0.08–0.39) | <0.001 |
| Charlson category 3-4 | 0.21(0.06–0.66) | 0.010 |
| Charlson category ≥5 | Ref | - |
