# Supplementary material for: Critical care utilisation, and outcomes of critically ill obstetric patients in India: a multicentre ICU registry study (2019–2024)
Source: Lancet Reg Health Southeast Asia. 2026 Aug 26;52:100845. doi: 10.1016/j.lansea.2026.100845 (PMC13544171; doi:10.1016/j.lansea.2026.100845)
Supplement: Supplementary file 4 [file mmc4.docx]

**Supplement Figure 1.** Cumulative incidence curves for non-obstetric versus obstetric patients. The y-axis is truncated at a cumulative incidence of 10% to improve visual clarity.

**Table 3.** Clinical characteristics of obstetric patients admitted to public versus private ICUs.

| **Characteristics** | **Obstetric patients admitted to Public ICUs**  (n = 157) | **Obstetric patients admitted to Private ICUs**  (n = 420) | **p-value** | **ASD** |
| --- | --- | --- | --- | --- |
| **Age** in years, median (Q1-Q3) | 27 (24-32) | 30 (26-33) | <0.001* | 0.328 |
| **Admission type**, n(%)  Non-operative  Post-operative | 28 (17.8)  129 (82.2) | 108 (25.7)  312 (74.3) | 0.060 | 0.190  0.190 |
| **APACHE II Score,** median (Q1-Q3) | 12 (8-19) | 8 (6-12) | <0.001* |  |
| **Invasive ventilation support** during the ICU stay, n(%) | 71 (45.2) | 53 (12.6) | <0.001* | 0.765 |
| **Cardiovascular support** during the ICU stay, n(%) | 64 (40.8) | 34 (8.1) | <0.001* | 0.821 |
| **RRT** during ICU stay, n(%) | 8 (5.1) | 11(2.6) | 0.221 | 0.132 |
| **ICU mortality**, n(%) | 10 (6.4) | 9 (2.1) | 0.017* | 0.219 |
| **APACHE II Predicted risk of death**  Median (Q1-Q3) | 0.12 (0.07-0.25) | 0.06 (0.04-0.12) | <0.001* | - |
| Mean (standard deviation) | 0.19 (0.16) | 0.11 (0.13) | § | 0.590 |
| **SMR with 95% CI** | 0.34 (0.16, 0.63) | 0.19 (0.08, 0.36) | 0.201 | - |
| **ICU length of stay** in days, median (Q1-Q3) | 3 (2-4) | 3 (2-3) | 0.024* | 0.106 |

APACHE, acute physiology and chronic evaluation; RRT, renal replacement therapy; SMR, standardized mortality rate; ICU, intensive care unit.

§P-value for the mean APACHE II predicted risk of death not computed due to skewed distribution, yet values are provided as this is utilized to compute the SMR.

*statistical significance at the 5% level (*p* < 0.05)

Supplement Table 1

Supplementary Table: Multivariable logistic regression analyses for ICU mortality

|  | Adjusted OR (95% CI) | p-value |
| --- | --- | --- |
| Model 1: Adjusted for APACHE II score and Charlson category |  |  |
| Obstetric vs non-obstetric | 0.47(0.28–0.74) | 0.002 |
| APACHE II Score | 1.13(1.12–1.14) | <0.001 |
| Charlson category 0 | 0.29(0.13–0.70) | 0.004 |
| Charlson category 1-2 | 0.19(0.08–0.47) | <0.001 |
| Charlson category 3-4 | 0.09(0.02–0.32) | <0.001 |
| Charlson category ≥5 | Ref | - |
| Model 2: Adjusted for age and Charlson comorbidity category |  |  |
| Obstetric vs non-obstetric | 0.42(0.25–0.65) | <0.001 |
| Admission age | 1.01(1.00–1.02) | 0.024 |
| Charlson category 0 | 0.18(0.09–0.40) | <0.001 |
| Charlson category 1-2 | 0.17(0.08–0.39) | <0.001 |
| Charlson category 3-4 | 0.21(0.06–0.66) | 0.010 |
| Charlson category ≥5 | Ref | - |
